# Inflammatory Duodenal Polyposis Associated with Primary Immunodeficiency Disease: A Novel Case Report

**DOI:** 10.1155/2017/6206085

**Published:** 2017-01-10

**Authors:** Irfan Ali Shera, Sheikh Mudassir Khurshid, Mohd Shafi Bhat

**Affiliations:** ^1^Department of Gastroenterology, Max Super Speciality Hospital, Saket, New Delhi 110 007, India; ^2^Department of General Surgery, Fortis Hospital, Mohali, Punjab 160 062, India; ^3^Department of Sports Medicine, Safdarjung Hospital, New Delhi, India

## Abstract

Agammaglobulinemia is a rare form of B-cell primary immunodeficiency disease characterized by reduced levels of IgG, IgA, or IgM and recurrent bacterial infections. Agammaglobulinemia is most commonly associated with diffuse nodular lymphoid hyperplasia. Duodenal polyps are a rare entity; however, due to wide use of esophagogastroduodenoscopy, incidental diagnosis of duodenal polyps appears to be increasing. Although inflammatory duodenal polyposis has been reported in the literature, its association with common variable immunodeficiency has not been reported till date to the best of our knowledge. We report a case of a 59-year-old male with chronic symptoms of agammaglobulinemia associated with inflammatory duodenal polyposis.

## 1. Introduction

Duodenal polyps are rare entity; however, due to wide use of esophagogastroduodenoscopy, incidental diagnosis of duodenal polyps seems to be increasing. Similarly, agammaglobulinemia is a form of B-cell primary immunodeficiency disease characterized by reduced levels of IgG, IgA, or IgM and recurrent bacterial infections with normal T-cell immunity in 60% of patients. Being the largest immune organ, gastrointestinal tract is affected by agammaglobulinemia in a wide spectrum of symptoms and signs. Herein, we present a case of inflammatory duodenal polyposis associated with agammaglobulinemia in a male patient admitted for evaluation of chronic diarrhoea in our hospital. To our knowledge, there is no such case in literature which depicts inflammatory duodenal polyposis seen in primary immunodeficiency disease. We emphasize the importance of considering agammaglobulinemia as a close differential diagnosis in a patient with duodenal polyposis by presenting this index case in a patient of chronic diarrhoea. Prevalence of duodenal polyps is nearly 0.3%–0.5% and 4.6% as revealed by various retrospective [1, 2] and prospective study [3], respectively, on esophagogastroduodenoscopy. Although duodenal polyps may be pedunculated in nature, these polyps are sessile and small, measuring 3 mm–10 mm. Most of polyps occurring in duodenum are nonneoplastic, which include ectopic gastric mucosa, hyperplastic polyps, and Brunner's gland hyperplasia. Inflammatory polyps contain ectopic gastric mucosa and are frequently present in duodenum [1, 2]. In the duodenal bulb, multiple polyps smaller than 10 mm do not need biopsy or treatment, whereas endoscopic surveillance and biopsy of duodenal polyps are important in patients with familial adenomatous polyposis [3], in which malignant transformation into adenomas or carcinoid tumours can be seen even if size is less than 10 mm and, hence, they need treatment.

## 2. Case Presentation

**Figure 1:**
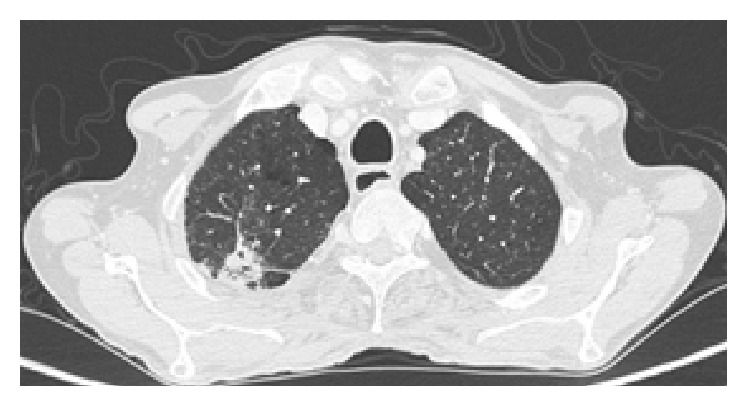
Fibrotic changes with focal pleural thickening in right apex with associated paraseptal emphysema suggestive of old healed lesion.

**Figure 2:**
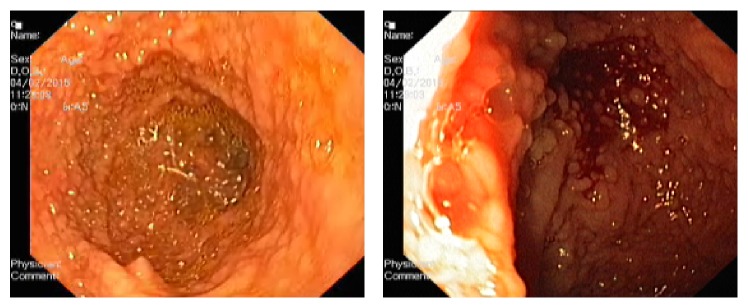
Multiple small polyps in D2 part of duodenum.

**Figure 3:**
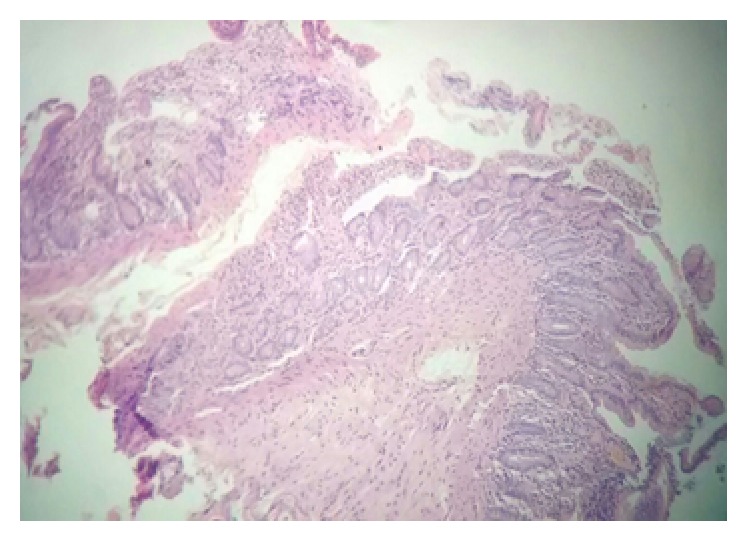
Duodenal mucosa shows preserved cryptovillus architecture and moderate lymphocytic infiltrate in the lamina propria along with a few neutrophils.

A 59-year-old male, Kurdish in origin, came to our hospital with history of recurrent chronic diarrhoea for last eighteen years. Each episode of diarrhoea was lasting for more than a month and used to get relieved with antibiotics. The patient had also history of pulmonary tuberculosis and recurrent sinopulmonary infections. We evaluated him thoroughly for his chronic diarrhoea. His stool examination showed cyst of* Giardia lamblia*. His routine blood examination reports were normal. His chest X-ray showed hyperinflated lungs; there was pleural thickening with inhomogeneous opacities suggestive of fibrosis in right upper zone. Ultrasonography of whole abdomen was normal. His contrast-enhanced computerized tomography of chest and abdomen revealed right apical pleural thickening and fibrosis in upper lobe and bronchial wall thickening, paraseptal emphysema, and subcentimetric mediastinal lymphadenopathy (Figure 1). Transbronchial nodal aspiration done was inconclusive. His esophagogastroduodenoscopy showed duodenal polyposis (Figure 2). His total colonoscopy and capsule endoscopy for small bowel were normal. His histopathological examination of duodenal specimen was consistent with features suggestive of inflammatory polyps (Figure 3). His 24-hour urinary protein was within normal limits. Immunological work-up was done. His human immunodeficiency viral serology was nonreactive. CD3, CD4, and CD8 counts were 1208/microlitre, 475/microlitre, and 707/microlitre, respectively. Immunoglobulin levels were IgG < 200 mg/dL (791–1643), IgA < 6 mg/dL (60–436), and IgM < 25 mg/dL (43–279). There was selective deficiency of B-cell (humoral) immunity.

## 3. Discussion

Agammaglobulinemia is a group of inherited immune deficiencies characterized by a low concentration of antibodies in the blood due to the lack of particular lymphocytes in the blood and lymph. Patients suffering from agammaglobulinemia have characteristic features of lack of B lymphocytes, decreased levels of immunoglobulins, chronic diarrhoea, granulomatous disease, recurrent infections, and joint involvement [2, 4, 5]. Agammaglobulinemia shows X-linked recessive pattern [6]. Initially, patients with agammaglobulinemia may present with clinical manifestations of loose motions, malabsorption, and loss of weight. Infectious cause of diarrhoea in agammaglobulinemia is seldom established [7]. Patients suffering from agammaglobulinemia have high prevalence of infectious, inflammatory, and malignant gastrointestinal disorders.

Agammaglobulinemia involves gastrointestinal tract like chronic-atrophic gastritis, giardiasis, sprue-like disorder with atrophied villi, inflammatory bowel disease, nonspecific intestinal malabsorption, nodular lymphoid hyperplasia, and pernicious anaemia [8, 9]. These gastrointestinal disorders seem to be due to defects in cellular immunity instead of deficiency of antibodies alone [9]. A broad spectrum of histologic changes ranging from marked atrophy of villi and increased lymphocytes in mucosa resembling celiac disease to nodular lymphoid hyperplasia can be seen in patients with agammaglobulinemia [10]. Almost every patient with agammaglobulinemia suffers from acute, recurrent, or chronic infections, especially conjunctivitis, otitis, sinusitis, bronchitis, and pneumonia [2]. However, effects of malabsorption such as deficiency of vitamins and electrolytes may be present in severe cases. Patients with agammaglobulinemia have malabsorption involving carbohydrates, dietary fats, vitamin B12, and folate.

Patients with history of recurrent bacterial infections presenting with gastrointestinal manifestations, specifically chronic diarrhoea, should be assessed for the possibility of agammaglobulinemia as any delay in the diagnosis and, hence, treatment can lead to significant morbidity and complications in patients with agammaglobulinemia. Treatment of gastrointestinal disorders in agammaglobulinemia with intravenous immunoglobulins alone may be ineffective in comparison to combination with immunomodulators such as azathioprine and 6-mercaptopurine, because most gastrointestinal manifestations of agammaglobulinemia appear to be due to defects in T-cell mediated immunity [1].

Prevalence of duodenal polyps is nearly 0.3%–0.5% and 4.6% as revealed by various retrospective [1, 2] and prospective studies [3], respectively, on esophagoduodenoscopy. Although duodenal polyps may be pedunculated in nature, these polyps are sessile and small, measuring 3 mm–10 mm. Most of polyps occurring in duodenum are nonneoplastic, which include ectopic gastric mucosa, hyperplastic polyps, and Brunner's gland hyperplasia. Inflammatory polyps contain ectopic gastric mucosa and are frequently present in duodenum [1, 2]. In the duodenal bulb, multiple polyps smaller than 10 mm do not need biopsy or treatment, whereas endoscopic surveillance and biopsy of duodenal polyps are important in patients with familial polyposis [3]. Malignant transformation into adenomas or carcinoid tumours can be seen in some duodenal polyps having size less than 10 mm and, hence, they need treatment. The clinical manifestations of nonneoplastic and neoplastic diseases of duodenum have not been identified so far. Duodenal polyps having hyperplastic (metaplastic) nature have seldom been identified. Data published on these polyps are restricted to case reports or small case reports [6, 8, 10]. They closely resemble hyperplastic polyps of the gastric type instead of the colonic type as they almost always arise in the context of ectopic mucosa of the stomach. Hyperplastic polyps of the duodenum seem to occur most frequently in patients with peptic ulcer disease or other gastric disorders [8] and can be associated with colonisation by* Helicobacter pylori *[7].

## 4. Conclusion

In conclusion, though there is no direct evidence of chronic diarrhea with inflammatory duodenal polyposis, the contributing factors for development of inflammatory polyps of duodenum with agammaglobulinemia in our patient may be due to recurrent infections of the gastrointestinal tract. We have described a rare case of inflammatory duodenal polyposis coexisting with agammaglobulinemia in a patient with chronic diarrhoea, and agammaglobulinemia should be considered in the list of differential diagnoses of inflammatory duodenal polyposis, especially when it is incidentally seen on esophagogastroduodenoscopy of a patient with chronic diarrhea such as in our case. The study needs further reports to establish cause and effect relationship between chronic diarrhea and inflammatory duodenal polyposis.
